# Targeted biopsy with or without systematic biopsy in PI-RADS-stratified prostate biopsy: a risk-adapted narrative review and clinical proposal

**DOI:** 10.3389/fonc.2026.1996528

**Published:** 2026-10-26

**Authors:** Ye Wu, Yun Wu, Qiang Lu, Guoou Xie

**Affiliations:** 1 Department of Urology, Hunan Aerospace Hospital, The Affiliated Aerospace Hospital of Hunan Normal University, Changsha, Hunan, China; 2 Department of Urology, The First Affiliated Hospital of Hunan Normal University/Hunan Provincial People’s Hospital, Changsha, Hunan, China

**Keywords:** biomarkers, PI-RADS, precision diagnosis, prostate cancer, PSA density, systematic biopsy, targeted biopsy, transperineal biopsy

## Abstract

Multiparametric magnetic resonance imaging (mpMRI)-guided targeted biopsy has transformed the diagnostic pathway for prostate cancer. However, the need for routine systematic biopsy in addition to targeted biopsy remains a subject of active controversy. This narrative review synthesizes evidence from prospective cohorts, meta-analyses, and randomized trials published between 2015 and 2026 to evaluate the diagnostic yield and risk trade-offs of targeted biopsy combined with systematic biopsy versus targeted biopsy alone across PI-RADS categories and to propose a risk-adapted biopsy protocol. The available evidence supports a strong dose-response relationship between PI-RADS score and the detection of clinically significant prostate cancer (csPCa). At the same time, this relationship is substantially modified by PSA density (PSAD), prostate volume, lesion size, lesion location, biopsy route, race, and biological markers. Combined targeted plus systematic biopsy still provides the highest overall csPCa detection rate, particularly for PI-RADS 4 lesions and small or equivocal lesions. However, targeted biopsy alone or targeted biopsy with limited perilesional systematic sampling may be cautiously considered for selected high-risk patients, especially those with PI-RADS 5 lesions, lesion size ≥1 cm, or high PSAD. However, this recommendation is based on indirect evidence from non-randomized studies and should be interpreted with caution pending results from ongoing randomized trials. In the indeterminate PI-RADS 3 category, no single imaging criterion is sufficient; PSAD thresholds, urinary biomarkers, and zone-specific risk estimates should be integrated to avoid unnecessary biopsy. A revised three-tiered decision framework is proposed: Selective Omission, conditional optimization, and standard coverage. The manuscript emphasizes that this is a structured narrative review and that de-escalation recommendations are not uniform across all PI-RADS 4–5 lesions. Future randomized trials should move beyond detection rates and evaluate adverse pathology, long-term oncologic outcomes, and patient-reported outcomes.

## 1 Introduction

### 1.1 Limitations of conventional systematic biopsy and the paradigm shift toward MRI-targeted biopsy

Multiparametric magnetic resonance imaging (mpMRI), as the most accurate imaging modality for detecting prostate cancer, has fundamentally transformed the diagnostic landscape of prostate cancer, enabling a paradigm shift from blind systematic sampling to visually guided precision biopsy (1). Conventional transrectal ultrasound-guided systematic biopsy (SB), lacking direct visualization of suspicious lesions, carries significant limitations—it not only tends to miss tumors located in the anterior prostate or apex but also leads to substantial overdiagnosis of clinically insignificant prostate cancer (ciPCa) (2). In contrast, MRI-targeted biopsy (TB), which identifies suspicious areas on pre-procedural imaging and performs targeted sampling, has significantly improved the detection rate of clinically significant prostate cancer (csPCa) (1, 3, 4). A randomized controlled trial incorporating 17,980 men demonstrated that, compared with systematic biopsy, MRI-targeted biopsy alone reduced the detection of clinically insignificant prostate cancer (Gleason score 3 + 3) by 54% (relative risk 0.46), while maintaining effective detection of clinically significant cancers (2, 5). Furthermore, MRI-based screening pathways demonstrated a 4.15-fold higher probability of detecting csPCa upon positive results compared with standard PSA screening pathways (6). Two-year follow-up data from the PRECISE trial further confirmed that the MRI-targeted biopsy strategy was non-inferior to the systematic biopsy strategy in csPCa detection, with 38% of men in the MRI arm avoiding initial biopsy (7). This transition from anatomical blind sampling to functional imaging-guided biopsy has established targeted biopsy as a cornerstone for improving diagnostic efficacy and reducing unnecessary medical interventions (8). The 2024 EAU guidelines now support targeted biopsy with perilesional sampling for PI-RADS ≥4 lesions rather than routine whole-gland systematic biopsy in all men. This recommendation reflects the growing willingness to individualize biopsy extent, but it also creates a new clinical challenge: clinicians must determine when systematic biopsy remains necessary (9).

### 1.2 The central role and evolution of the PI-RADS in biopsy decision-making

Since its introduction in 2012, the Prostate Imaging Reporting and Data System (PI-RADS) has become a standardized tool for radiologists and urologists in prostate cancer clinical management and biopsy decision support (10). The current PI-RADS v2.1 is widely applied to guide biopsy procedures, with scores demonstrating a clear dose-response relationship with csPCa detection risk. Large-scale multicenter data show that the absolute risk of Grade Group ≥2 disease for PI-RADS 4 and 5 lesions ranges from 23%-68% and 49%-87%, respectively (11, 12). In a prospective cohort of 1,215 men, the csPCa detection rates for PI-RADS 2, 3, 4, and 5 lesions were 4.4%, 15%, 39%, and 74%, respectively (12). Despite the critical role of PI-RADS in standardizing reporting, its limitations persist—there is a two-fold variation across centers in the probability of assigning PI-RADS 4 or 5, and diagnostic performance is influenced by reader experience and anatomical zone (10, 11, 13). Therefore, PI-RADS category should not be used in isolation. It is the foundation for risk-based decision-making but requires integration with clinical and biological variables.

### 1.3 The current controversy: overdiagnosis risk of combined biopsy versus missed diagnosis risk of targeted biopsy alone

Although MRI-targeted biopsy offers clear advantages, controversy remains regarding whether systematic biopsy should be routinely combined (TB+SB). On one hand, combined biopsy improves overall detection rates but introduces overdiagnosis risk. Studies show that the detection rate of ISUP Grade Group 1 prostate cancer is significantly higher in the combined biopsy group compared with targeted biopsy alone (OR 1.16) (14), and in screening trials, combined biopsy detects more indolent cancers (15). On the other hand, targeted biopsy alone carries non-negligible missed diagnosis risks. A multi-institutional study reported that 17% of clinically significant cancers would be missed if targeted biopsy alone were performed (16); another retrospective analysis found that 18% of patients were diagnosed with csPCa only through sampling outside the visible lesion (17). Reasons for missed diagnosis include targeting errors (51.2%), MRI-invisible lesions (40.5%), and radiologist oversight (7.1%) (18). Particularly when planning focal therapy, targeted biopsy alone may result in 59% of patients having additional tumors in unsampled quadrants, leading to misclassification as eligible for focal therapy (19). However, evidence also supports the safety of omitting systematic biopsy in specific high-risk populations—for example, when PSAD ≥0.2 ng/mL/cm³, the incremental csPCa detection yield of systematic biopsy over targeted biopsy alone is only 2% (20). This trade-off between “avoiding overdiagnosis” and “preventing missed diagnosis” underscores the inadequacy of the current “one-size-fits-all” combined biopsy approach.

### 1.4 Purpose and review questions

Given the above controversies, there is an urgent need to establish a risk-stratified precision biopsy strategy to replace the undifferentiated combined biopsy model. Existing studies have preliminarily validated the feasibility of a stratified approach: for patients with PI-RADS 3 and PSAD <0.13 ng/mL/cm³, biopsy may be avoided to reduce unnecessary overdiagnosis (21); for patients with high PSAD, systematic biopsy may be safely omitted (20); and perilesional sampling may replace whole-gland systematic biopsy, reducing non-significant cancer detection without compromising csPCa detection (14, 22).

This review addresses four specific questions:

1. Across PI-RADS 3, 4, and 5, what is the incremental csPCa yield of systematic biopsy over targeted biopsy alone?
2. Which men can safely be offered targeted biopsy alone or limited systematic sampling?
3. Which men still require standard combined biopsy for accurate grading, staging, and treatment planning?
4. How should PSAD, prostate volume, lesion characteristics, biomarkers, and biopsy route modify the biopsy strategy?

The final objective is to propose a tiered, risk-adapted biopsy protocol rather than a uniform all-or-nothing approach.

## 2 Search strategy and evidence synthesis

This is a narrative review. We searched PubMed, Embase, Cochrane Library, and Web of Science for English-language articles published from January 2015 to August 2026. Search terms included “prostate cancer,” “clinically significant prostate cancer,” “PI-RADS,” “targeted biopsy,” “systematic biopsy,” “combined biopsy,” “PSA density,” “transperineal biopsy,” “SelectMDx,” “ExoDx,” “Stockholm3,” and “risk stratification.” We included retrospective and prospective cohort studies, meta-analyses, and randomized controlled trials. Case reports, pediatric studies, and unpublished conference abstracts were excluded. No formal statistical pooling was performed. We prioritized evidence according to design: meta-analyses, randomized trials, prospective cohorts, and large multicenter registries were given greater weight than single-center retrospective series. This review was not registered in PROSPERO, and a PRISMA checklist was not applicable because no systematic review was attempted.

### 2.1 Transparency and limitations of the narrative approach

The initial search yielded approximately 1,847 records; after removal of duplicates, 1,203 records remained. After title/abstract screening, 312 full-text articles were assessed, and 92 studies were included in the final narrative synthesis (**Supplementary Table S1**). A simplified evidence flow diagram is presented in **Figure 1**. The authors explicitly acknowledge the selective nature of this evidence synthesis and the potential for selection and interpretation bias inherent to the narrative format. The search strategy was designed for comprehensive coverage rather than exhaustive systematic retrieval, and no formal quantitative pooling was performed. A PRISMA-style flow diagram (**Figure 1**) and evidence map (**Supplementary Table S1**) are provided to enhance transparency.

**Figure 1:**
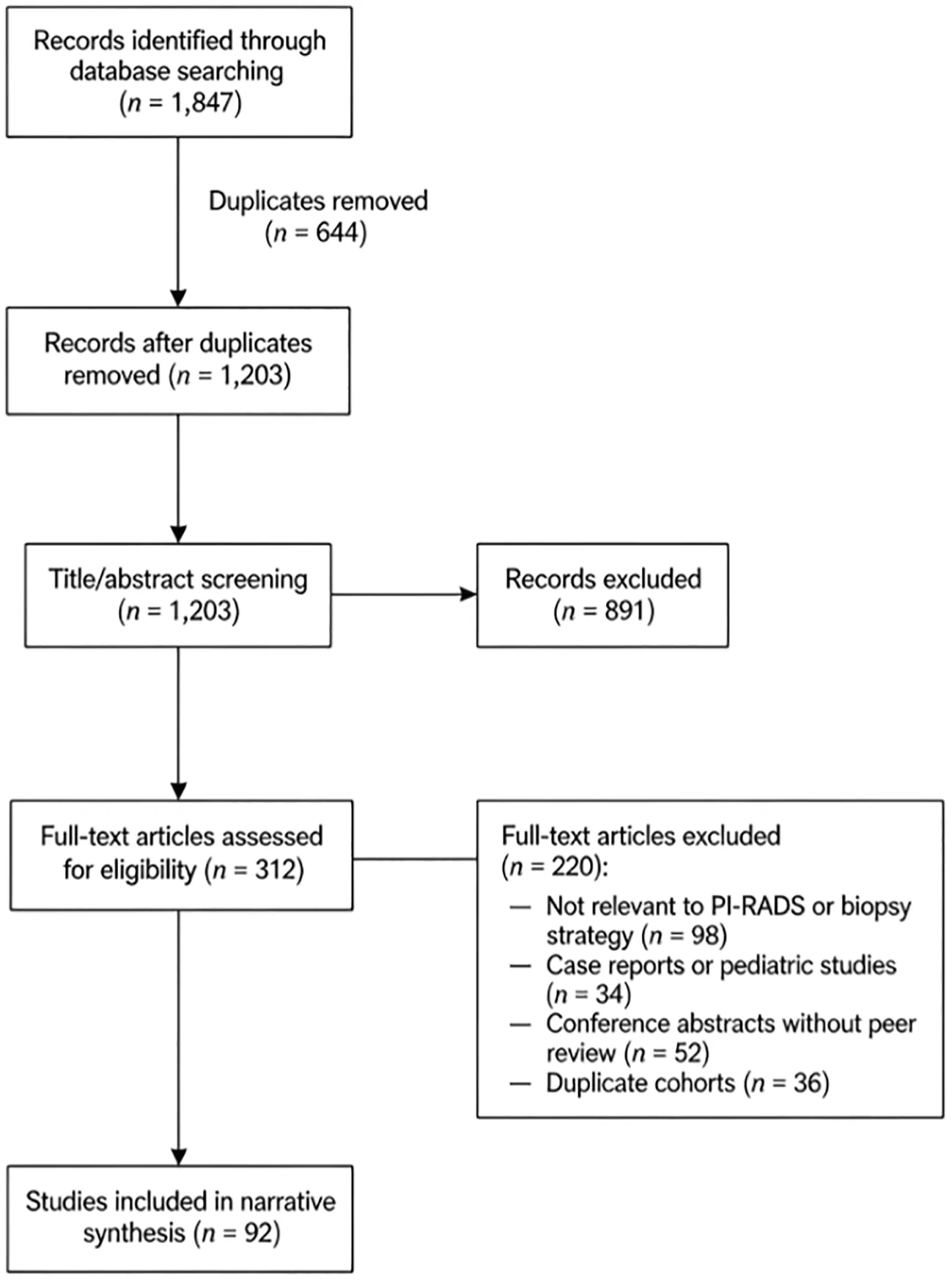
Simplified evidence identification and selection flow diagram.

## 3 Diagnostic performance of the PI-RADS system and the basis for risk assessment

Since its introduction in 2012, the PI-RADS system has become the core standardized tool for mpMRI assessment and biopsy decision-making in prostate cancer (10). With successive version updates, the system demonstrates high sensitivity for csPCa detection but faces challenges of limited specificity. A systematic review and meta-analysis of 70 studies encompassing 13,330 patients showed that at a patient-level threshold of PI-RADS ≥3, pooled sensitivity reached 96% (95% CI: 95%-98%) but specificity was only 43% (95% CI: 33%-54%); when the threshold was raised to ≥4, sensitivity decreased to 89% (95% CI: 85%-92%) while specificity improved to 66% (95% CI: 58%-74%) (23). Another meta-analysis of 117 studies confirmed similar trends (24). These findings indicate that anatomical zone heterogeneity must be fully considered when interpreting PI-RADS scores. These test characteristics support the use of PI-RADS as a risk stratification tool rather than a definitive diagnostic marker.

### 3.1 Diagnostic accuracy comparison between PI-RADS v2 and v2.1 in different anatomical zones (PZ/TZ)

The PI-RADS system exhibits inherent anatomical differences in diagnostic accuracy between the peripheral zone (PZ) and transition zone (TZ), which, although improved from v2 to v2.1, remain significant. Studies show that TZ lesions are more prone to false-positive results than PZ lesions (OR = 1.74, P = 0.006), primarily due to signal overlap between benign prostatic hyperplasia nodules and malignancy on DWI sequences (25). In PI-RADS v2.1 application, the csPCa detection rate for TZ 2 + 1-type lesions (DWI positive + DCE positive) was 12% (95% CI: 7%-19%), while for 3 + 0-type lesions (DWI positive + DCE negative) it was 19% (95% CI: 12%-29%), with no statistically significant difference (P = 0.12) (23). For TZ nodules upgraded to PI-RADS 3, the csPCa detection rate was significantly lower than for non-upgraded lesions (4% vs 20%, P = 0.02), indicating that DCE sequence plays a limited role in risk stratification of TZ lesions (26). In contrast, quantitative ADC values have greater value in classifying PZ PI-RADS 3 lesions, with an ADC cutoff of 0.897×10⁻³ mm²/s effectively differentiating benign from malignant lesions (27). Lesion location itself is an independent predictor, with TZ lesions showing lower odds of csPCa detection compared with PZ lesions (OR 0.34, P = 0.009) (28).

### 3.2 Reader agreement and standardization challenges in image interpretation

The subjectivity inherent in PI-RADS scoring, leading to inter-reader variability, is a critical factor affecting the stability of biopsy decisions. Studies demonstrate that radiologist experience directly influences diagnostic accuracy—those with lower annual reading volumes or without specialized training are more prone to false-negative results (P<0.001), and an annual volume exceeding 50 reads significantly reduces false-negative risk (29). In multicenter studies, the probability of assigning PI-RADS 4 or 5 varied two-fold across centers (adjusted heterogeneity p<0.001), resulting in absolute csPCa risks for the same score ranging from 23% to 68% for PI-RADS 4 and 49% to 87% for PI-RADS 5 (11). Artificial intelligence-assisted techniques show promise in improving this situation. An international multicenter non-inferiority confirmatory study found that AI systems achieved an AUROC of 0.91 for csPCa detection, significantly outperforming radiologists at 0.86 (p<0.0001), while maintaining the same sensitivity (89.4%) and reducing false positives by 50.4% (30). Combined AI-radiologist reading further improved sensitivity, particularly in low-risk and indeterminate PI-RADS lesions. Nevertheless, current AI sensitivity at the lesion level (80%) remains lower than that of radiologists (93%) (31, 32), suggesting AI is better suited as a quality assurance tool rather than a complete replacement for human reading.

### 3.3 Dose-response relationship between PI-RADS category and csPCa detection rate

A clear and robust dose-response relationship exists between PI-RADS category and csPCa detection rate, forming the cornerstone of biopsy decision guidance. Large-scale meta-analysis data show csPCa detection rates by PI-RADS grade: 6% for grade 1, 5% for grade 2, 19% for grade 3, 54% for grade 4, and 84% for grade 5 (23). Prospective cohort studies further refined these estimates, with PI-RADS 1–5 corresponding to csPCa rates of 0%, 9%, 14%, 37%, and 77%, respectively, with statistically significant differences between adjacent grades (P<0.001) (26). Mixed-effects meta-analysis confirmed that PI-RADS 4 (β=7.82, P = 0.045) and PI-RADS 5 (β=23.18, P<0.001) were significantly positively associated with csPCa risk, whereas PI-RADS 3 showed no significant linear association (β=-4.08, P = 0.19), highlighting the special “gray zone” nature of score 3 (33). Beyond detection rates, PI-RADS score is also closely associated with long-term prognosis—PI-RADS 5 is significantly associated with prostate cancer-specific mortality (adjusted HR 18.4) (34). In patients on active surveillance, baseline PI-RADS ≥4 was significantly associated with an increased risk of reclassification to higher-grade prostate cancer on follow-up biopsy (HR 2.3, P < 0.001) (35). However, this dose-response relationship is modulated by clinical variables such as PSAD, which is the only clinical variable significantly associated with csPCa in multivariate models (β=15.50, P<0.001), and combining PI-RADS with PSAD significantly improves risk stratification performance (33).

### 3.4 Reassessment of PI-RADS diagnostic value in special populations

In patients with prior negative prostate biopsies, the diagnostic performance of PI-RADS scores shifts significantly. Studies show that csPCa detection rates in the prior-negative biopsy group are significantly lower than in biopsy-naïve patients at the same PI-RADS grade (e.g., PI-RADS 3: 5.2% vs 15.4%), and csPCa detection in prior-negative patients with PI-RADS ≤3 is only 5.3% (36). This suggests that tissue trauma and inflammation may confound image interpretation, shifting the correspondence between scores and pathological outcomes. Racial differences also indicate the impact of biological context on PI-RADS performance—Black men have higher odds of csPCa in PI-RADS 3/4 lesions compared with White men (OR 1.20, P = 0.004), while Asian men showed 0% csPCa detection in PI-RADS 3 lesions, significantly lower than other ethnicities (P = 0.003) (37). For carriers of germline mutations, MRI screening (PI-RADS ≥3) achieved 100% sensitivity and 100% negative predictive value for csPCa detection (38). These findings collectively suggest that universal PI-RADS thresholds may not apply in special populations with altered anatomical structure or distinct biological characteristics, and customized calibration coefficients should be established in clinical practice (39).

To sum up, PI-RADS performance varies with: reader experience; MRI field strength and protocol quality; lesion location; prostate volume; prior biopsy status; race and population-specific cancer prevalence. These modifiers explain why PI-RADS alone does not produce a fixed probability of csPCa and why clinical decision models should include PSAD, lesion size, and other variables.

## 4 The value of adjunctive parameters in biopsy decision optimization

### 4.1 Evidence for PSAD combined with PI-RADS to improve diagnostic specificity

PSAD has been established as the only clinical variable independently associated with csPCa aside from PI-RADS 5 (33). When PI-RADS scores fall into the gray zone or low-risk categories, incorporating PSAD significantly improves diagnostic specificity and reduces unnecessary biopsies (33). A mixed-effects meta-analysis of 72 studies showed that avoiding biopsy in patients with PI-RADS ≤3 and PSAD <0.10 ng/mL/cm³ could reduce unnecessary procedures by 30% while maintaining 97% sensitivity. For the most controversial PI-RADS 3 subgroup, systematic review data show that at a PSAD threshold of 0.15 ng/mL/cm³, specificity for csPCa diagnosis reached 0.69, increasing to 0.82 when the threshold was raised to 0.2 ng/mL/cm³ (40). PSAD diagnostic performance is also significantly influenced by lesion anatomical location (41). In prospective cohort studies, adopting a post-MRI PSAD threshold of ≥0.20 ng/mL/cm³ enabled 29% of PI-RADS 3 patients to avoid negative biopsies, missing only 1.3% of GG≥2 cancers (42). The MR-PROPER study further demonstrated that in PI-RADS 3 patients, using a PSAD threshold of ≥0.20 ng/mL/cm³ rather than the guideline-recommended 0.10 ng/mL/cm³ significantly improved the benefit-risk balance. The risk-stratification pathway of “MRI + PSAD + f/tPSA” developed by Jin et al. achieved 40% biopsy avoidance with 94% csPCa detection sensitivity and the lowest negative biopsy rate (43). In a subset of patients with high suspicion of prostate cancer, the combined PI-RADS and PSAD prediction model yielded a 98.2% PPV for csPCa in biopsy-naïve radical prostatectomy specimens, reinforcing its utility for accurately identifying high-risk candidates (44).

### 4.2 Stratification role of prostate volume thresholds in avoiding unnecessary biopsy

Prostate volume is significantly negatively correlated with prostate cancer probability, making it an important risk stratification tool (45). Studies demonstrate that PV modifies the risk prediction of PSAD for csPCa: when PV <45 mL, a PSAD of 0.10 ng/mL/cm³ corresponds to a csPCa risk of 37%; when PV increases to 60–100 mL, the risk drops to 15% at the same PSAD level (46). For PI-RADS 3 lesions, when PV <40 mL, csPCa risk exceeds 10% regardless of PSAD value, suggesting that patients with small glands require more aggressive evaluation; conversely, for PV >40 mL, the PSAD threshold to achieve 10% csPCa risk lies between 0.10-0.15 ng/mL/cm³ (46). These data indicate that PSAD must not be interpreted in isolation when formulating biopsy strategies—dynamic threshold adjustment based on prostate volume is essential (47).

### 4.3 Safe monitoring boundaries for PI-RADS 1–2 patients

Although PI-RADS 1–2 is generally considered negative, csPCa missed diagnosis risk persists in specific clinical contexts. Studies show that in patients with MRI-negative (PI-RADS 1-2) and PSAD <0.15 ng/mL/cm³, only 3% were reclassified as GG≥2 during active surveillance follow-up, and 0.6% as GG≥3, suggesting that protocol-mandated repeat biopsy can be safely avoided in this population (48). However, for PI-RADS 1–2 patients with elevated PSAD, sole reliance on MRI may lead to misjudgment (49). The Göteborg 2 screening trial showed that 89% of prior MRI-negative men remained negative in the second round, and a 2-year interval appears safe in MRI-negative men (50). Therefore, biopsy should not be uniformly exempted in PI-RADS 1–2 patients—comprehensive assessment incorporating PSAD, race, and novel molecular imaging techniques is warranted (23, 24, 33, 51, 52).

### 4.4 Comprehensive efficacy of multiparameter models in reducing unnecessary biopsy

Single indicators often have limitations in guiding biopsy decisions; integrating multiple parameters including PI-RADS, PSAD, and PV demonstrates superior comprehensive performance. A study based on STHLM3-MRI data showed that the optimal model incorporating age, PV, Stockholm3 density, and MRI lesion volume ratio could reduce biopsies by 33% while maintaining 98% sensitivity for ISUP≥2 cancers (53). Another multicenter study evaluating prostate health index density combined with PI-RADS found this combination achieved an AUC of 0.85, outperforming PI-RADS combined with PSAD (0.81) (12). AI-assisted multimodal models also confirmed the advantage of parameter integration, an early-fusion model incorporating deep learning-based suspicion and clinical variables (PSA, PV, age) attained an AUC of 0.77 on external validation, which was significantly superior to models relying solely on clinical data or imaging scores alone (54). These findings consistently indicate that constructing multiparameter risk prediction models incorporating imaging scores, PSAD, and PV is a key pathway to transitioning from “comprehensive coverage” to “precision de-escalation” biopsy strategies.

## 5 Comparative evidence by PI-RADS category

### 5.1 PI-RADS 4–5 lesions

In the PI-RADS 4–5 high-risk population, the core controversy regarding whether to retain systematic biopsy lies in balancing diagnostic efficacy against overtreatment risk. Current evidence suggests that while combined biopsy remains the standard recommended by many guidelines, targeted biopsy alone demonstrates non-inferiority under specific conditions.

#### 5.1.1 Evidence supporting de-escalation of systematic biopsy in selected PI-RADS 4–5 patients

A meta-analysis of 21 studies showed no statistically significant difference in overall csPCa (ISUP≥2) detection between targeted biopsy with regional biopsy and standard targeted with systematic biopsy (46.1% vs 44.2%; OR 1.07, p=0.07), nor for ISUP≥3 high-grade tumors (55). The recently completed multicenter paired non-inferiority trial (NCT06584279) included 564 biopsy-naïve men (PI-RADS ≥4 or PI-RADS 3 with PSAD ≥0.15 ng/mL/cm³) and demonstrated that targeted biopsy with ipsilateral systematic biopsy (TB+iSB) was non-inferior to standard combined biopsy for csPCa detection (40.78% vs 42.38%; difference -1.6%, 95% CI [-2.69, -0.5]), with slightly lower non-significant cancer detection. A prospective cohort study showed that when PSAD ≥0.2 ng/mL/cm³, the incremental csPCa detection yield of systematic biopsy over MRI-targeted biopsy alone was only 2% (95% CI: 0.4%-5.9%) (20). In PI-RADS 5 lesions and PI-RADS 3–4 lesions ≥1 cm, targeted biopsy alone detected 72.5% of prostate cancers, with only 1.4% csPCa upgrading (56). However, a 2026 network meta-analysis published in Nature Communications (61 studies, ~26,000 men) concluded that omitting systematic biopsy in PI-RADS 5 lesions is not supported by current evidence (57). This finding directly challenges earlier de-escalation recommendations and necessitates a more cautious approach. Consequently, we have downgraded the Tier 1 recommendation for PI-RADS 5 and explicitly grade the evidence supporting systematic biopsy omission as Low (57).

#### 5.1.2 Evidence supporting continued combined biopsy

Despite the strong performance of targeted biopsy alone in the high-risk group, systematic biopsy retains unique value in correcting grade underestimation and detecting MRI-invisible lesions. A study of 1,236 patients showed that combined biopsy detected csPCa in 33.3%, significantly higher than targeted biopsy alone (27.9%); more importantly, targeted biopsy led to upgrading to ≥GG2 in 9.8% of patients with negative systematic biopsy and in 23.6% of GG1 patients, confirming the risk-stratification correction effect of combined sampling (58). Another study found that 22% of patients had mpMRI-unrecognized Gleason ≥7 disease, and 4% had ≥8 disease—relying solely on mpMRI would miss over 20% of csPCa (59). For <1 cm PI-RADS 3–4 lesions, combined biopsy detection (51.2%) exceeded targeted biopsy alone (48.0%), suggesting that systematic biopsy plays a more critical supplementary role in small-volume high-risk lesions (56).

#### 5.1.3 Clinical synthesis for PI-RADS 4–5

The evidence supports a differentiated approach: 1) PI-RADS 5: Targeted biopsy alone or targeted biopsy with ipsilateral/perilesional systematic biopsy can be considered, especially if the lesion is ≥1 cm or PSAD is high (12, 17, 20, 60). 2) PI-RADS 4: Routine omission of systematic biopsy cannot be recommended for most patients (60–62). Limited systematic biopsy may be acceptable if markers are reassuring, but standard combined biopsy is still safer for staging and grade concordance (60, 61). 3) Regardless of PI-RADS category, systematic sampling should be directed to the ipsilateral and perilesional zones rather than the contralateral apex and base when de-escalation is being attempted (17, 63). Updated evidence from a 2026 network meta-analysis (61 studies, ~26,000 men) concluded that targeted biopsy alone remains insufficient, and omitting systematic biopsy in PI-RADS 5 lesions is not supported by current evidence (57). This finding directly challenges earlier de-escalation recommendations and necessitates a more cautious approach. Consequently, we have downgraded the Tier 1 recommendation for PI-RADS 5 and explicitly grade the evidence supporting systematic biopsy omission as Low (57).

#### 5.1.4 Targeted biopsy plus perilesional biopsy (TB+PLB) as the preferred strategy for PI-RADS 4-5

Emerging evidence from multiple prospective trials and consensus statements now supports TB+PLB as the standard strategy for PI-RADS 4–5 lesions (64). The MIRAGE study (2026) (22)provided the first prospective evidence that when TB+PLB is deliberately incorporated into the biopsy protocol, distant systematic biopsies add minimal diagnostic benefit and should be omitted. The ProBIOPSY international consensus (2026) (65) concluded that targeted plus perilesional biopsy within a 10-mm penumbra is sufficient for most diagnostic and local treatment planning decisions. The PERI-PRO non-inferiority RCT (2025) demonstrated that TPLBx achieved non-inferior csPCa detection compared with standard combined biopsy while significantly improving patient safety. These findings are further supported by two additional RCTs (PMID: 40760321 (66) and PMID: 38158249 (67)) showing that TB+iSB is non-inferior to TB+SB. We therefore recommend TB+PLB within a 10-mm perilesional penumbra as the preferred biopsy strategy for PI-RADS 4–5 lesions, with standard whole-gland systematic biopsy reserved for Tier 3 indications.

#### 5.1.5 Biopsy-free strategies for PI-RADS 4-5

While biopsy remains the reference standard for PI-RADS 4–5 lesions, emerging evidence supports biopsy-free strategies in carefully selected patients. Wang et al. (2025) (68) developed a biopsy-free nomogram incorporating Prostate Health Index (PHI) and PSMA SUVmax that could potentially reduce biopsies by 92.41% while missing only 1.53% of csPCa cases in biopsy-naïve men with PI-RADS ≥4 lesions. A related model combining PSMA-PET and PSA demonstrated high diagnostic accuracy and potential to avoid unnecessary biopsy in men with PI-RADS ≤3 MRI. These strategies should be considered in Tier 2 (Conditional Optimization Zone) for patients with negative confirmatory testing, but do not replace TB+PLB for patients who proceed to biopsy.

### 5.2 PI-RADS 3 indeterminate lesions

PI-RADS 3 lesions represent the diagnostic “gray zone,” with heterogeneity making biopsy decisions highly challenging. Sole reliance on PI-RADS 3 as a biopsy indication leads to numerous unnecessary procedures; incorporating adjunctive parameters for risk stratification is key to optimizing management in this group.

#### 5.2.1 PSAD-based triage

PSAD is the best-evidenced adjunctive tool for triaging PI-RADS 3 patients. In an analysis of 790 PI-RADS 3 cases, Mjaess et al. confirmed that PSAD was the only meaningful predictor, with thresholds <0.13 (especially <0.09) safely avoiding biopsy (69). CHAID decision tree analysis identified 0.13 ng/mL/cm³ as the key cutoff—below this value, especially below 0.09 ng/mL/cm³, csPCa detection rates dropped to 1% and 0.5%, respectively (21). Anatomical zone-specific analysis further showed that when PZ PSAD <0.05 ng/mL/cm³ or TZ PSAD <0.13 ng/mL/cm³, csPCa detection rates were below 10% (41). The MR-PROPER study showed that post-MRI PSAD ≥0.20 ng/mL/cm³ missed only 1.3% of GG≥2 cancers (42). However, thresholds are not fixed across populations or volume measurement methods. A critical technical issue is that MRI-derived prostate volumes are generally smaller than TRUS-derived volumes (70). Because PSAD is PSA divided by prostate volume, using an MRI-derived volume produces a higher PSAD than using a TRUS-derived volume for the same PSA value. This can push patients across the threshold and produce misclassification. Therefore, any recommended PSAD threshold must specify the volume source and should be locally calibrated (71, 72). This issue is especially important in Asian populations, where prostate volumes and normal PSA levels may differ (73, 74), but race-specific threshold data are limited (37, 75). PSAD thresholds must be interpreted in a scenario-specific manner. For biopsy-naïve men with PI-RADS 3 lesions: (1) transperineal route: PSAD <0.12 ng/mL/cm³ may safely avoid biopsy (csPCa risk <5%); (2) transrectal route: PSAD <0.15 ng/mL/cm³ may be considered for deferral; (3) peripheral zone: PSAD <0.05 ng/mL/cm³ yields csPCa detection <10%; (4) transition zone: PSAD <0.13 ng/mL/cm³ yields csPCa detection <10%. The MR-PROPER study demonstrated that a post-MRI PSAD threshold of ≥0.20 ng/mL/cm³ in PI-RADS 3 patients avoided 29% of unnecessary biopsies while missing only 1.3% of GG≥2 cancers. The acceptable miss rate for de-escalation should be <5% for GG≥2 and <10% for GG≥3. Buhas et al. (2026) further demonstrated that PSAD-based omission of systematic cores is feasible in PI-RADS 3 lesions, but the specific thresholds depend on biopsy route and targeted core number.

#### 5.2.2 Biomarker-assisted triage

For PI-RADS 3 patients with borderline PSAD, urinary biomarkers can refine risk (53). 1) SelectMDx: In PI-RADS 3 lesions, using a positive SelectMDx score to select biopsy would detect 81.3% of prostate cancers and 85.7% of csPCa in that category, while avoiding 42–45.8% of biopsies. In this subgroup, SelectMDx showed complementary value alongside PSAD in the PI-RADS 3 subgroup, but head-to-head comparative data are limited. 2) ExoDx Prostate IntelliScore (EPI): This urinary exosomal assay has high negative predictive value and can reduce unnecessary biopsy. In selected pathways, ExoDx plus MRI avoids about 43% of biopsies while missing 4.8% of high-grade cancers; 3) Stockholm3: It reduces unnecessary biopsies by 32–45% in general cohorts, but PI-RADS 3-specific comparative data are lacking (53). It should not yet be considered superior to SelectMDx or ExoDx in this subgroup. Operational roles, trigger points, evidence levels, accessibility, and cost of biomarkers are summarized in **Table 1**.

**Table 1:** Operational roles of biomarkers in prostate biopsy decision-making.

Biomarker	Trigger point	Clinical scenario	Evidence level	Accessibility	Cost
SelectMDx	Positive (score above validated cutoff)	PI-RADS 3, borderline PSAD	Moderate	Limited; mainly US/EU	High
ExoDx Prostate IntelliScore (EPI)	>15.6	PI-RADS 3, PSAD 0.10–0.15	Moderate	Limited; mainly US	High
Stockholm3	≥11% (pre-MRI) or ≥15% (post-MRI)	Pre-MRI screening, general population	Moderate	Limited; mainly Nordic countries	Moderate
PHI density	>0.30	PI-RADS 3, equivocal PSAD	Low-Moderate	Widely available	Moderate
PSMA-PET	SUVmax threshold (varies)	PI-RADS 3–4, biopsy-free strategy	Low	Limited; specialized centers	Very High

PHI, Prostate Health Index; PSMA, prostate-specific membrane antigen; SUVmax, maximum standardized uptake value. Evidence levels assigned using GRADE methodology.

#### 5.2.3 Core reduction in PI-RADS 3 patients who require biopsy

For PI-RADS 3 patients who must undergo biopsy, optimizing the number and extent of cores is key to minimizing trauma. The SEXTANT-PRO randomized controlled trial (NCT06684652) of 506 biopsy-naïve men demonstrated that targeted biopsy with six-core sextant systematic biopsy (TB + 6SB) was non-inferior to targeted biopsy with 12-core extended systematic biopsy for csPCa detection (54.3% vs 54.8%; difference -0.5%, 95% CI [-9.2%, 8.3%]), while significantly improving patient comfort and quality of life (55). Data show that the most relevant pathological diagnosis of the index lesion can be determined within 3 targeted cores, covering 97% of patients (76). In terms of spatial extent, perilesional biopsy within 10 mm of the lesion achieves comparable results to whole-gland systematic biopsy (63).

#### 5.2.4 Surveillance and monitoring strategies

For low-risk PI-RADS 3 patients, active surveillance and delayed biopsy strategies demonstrate favorable safety. The RAPID diagnostic pathway study showed that in PI-RADS 3 patients with PSAD <0.12 ng/mL, PSA monitoring rather than immediate biopsy achieved 91.9% 5-year GG≥2 cancer diagnosis-free survival (77). Ga-PSMA-11 PET-CT achieved 93% negative predictive value for excluding csPCa in PI-RADS 3 lesions (78). The PRIMARY score combined with PSAD strategy achieved 0% csPCa miss rate while avoiding 69.6% of biopsies in external validation (79).

### 5.3 PI-RADS 1–2 lesions

In PI-RADS 1–2 patients, routine biopsy is not recommended unless other indicators are present, including high PSAD, abnormal digital rectal examination, persistent rising PSA, family history, or high genomic risk (40, 79). For these patients, follow-up with PSA kinetics and repeat imaging may be more appropriate than immediate biopsy (25, 78).

### 5.4 Lesion characteristics and biopsy route

Lesion anatomical location and morphological features are key variables for optimizing biopsy approaches in high-risk groups. For anterior and apical lesions, the transperineal (TP) route shows clear advantages—meta-analysis demonstrates that TP approach yields significantly higher csPCa detection for anterior (OR 2.17, p<0.001) and apical (OR 1.86, p=0.01) lesions compared with transrectal approach (80). For TZ lesions, PI-RADS v2.1 assessment shows that TZ lesions are more prone to false-positive results (25). Lesion size also informs decision-making—for PI-RADS 3–4 lesions ≥1 cm and all PI-RADS 5 lesions, targeted biopsy alone is sufficient; for <1 cm lesions, systematic biopsy is recommended to reduce missed diagnosis and grade underestimation risk (56). Racial differences also warrant consideration (37).

#### 5.4.1 How biopsy route modifies biopsy strategy

The biopsy route significantly modifies the diagnostic yield and safety profile of de-escalation strategies. For transperineal biopsy: (1) anterior and apical lesions show significantly higher csPCa detection compared with transrectal approach (OR 2.17 and 1.86, respectively); (2) the incremental value of systematic biopsy over TB+PLB is lower, supporting de-escalation in Tier 1; (3) infection risk is substantially reduced, favoring this route when systematic biopsy is omitted. For transrectal biopsy: (1) posterior lesions are adequately sampled; (2) the incremental value of systematic biopsy is higher, favoring more conservative de-escalation. We therefore recommend that route-specific recommendations be incorporated into all tiers: Tier 1 (Selective Omission) favors transperineal TB+PLB; Tier 2 (Conditional Optimization) favors transperineal route when feasible for more precise perilesional sampling; Tier 3 (Standard Coverage) may use either route depending on lesion location.

### 5.5 Clinical scenario-specific considerations

#### 5.5.1 Biopsy-naïve patients

Higher baseline csPCa risk at any PI-RADS category. TB+PLB within 10-mm penumbra is preferred for PI-RADS 4-5. For PI-RADS 3, PSAD-based triage is most useful in this setting.

#### 5.5.2 Prior-negative biopsy patients

csPCa detection rates are significantly lower than in biopsy-naïve patients at the same PI-RADS grade (e.g., PI-RADS 3: 5.2% vs 15.4%). TB alone may be sufficient for PI-RADS 3 with PSAD <0.13 ng/mL/cm³. Combined biopsy should be reserved for PI-RADS 4–5 with high PSAD or suspicious DRE.

#### 5.5.3 Active surveillance patients

Baseline PI-RADS ≥4 is associated with increased reclassification risk (HR 2.3, P<0.001). Protocol-mandated repeat biopsy may be safely avoided in MRI-negative (PI-RADS 1-2) patients with PSAD <0.15 ng/mL/cm³, as only 3% were reclassified as GG≥2 during follow-up. However, an unsuspicious MRI at 6 years alone is insufficient to omit the 6-year AS biopsy under current guidelines. Combining MRI findings, PSAD, and prior negative AS biopsies is a promising strategy to tailor surveillance intensity.

## 6 Constructing a risk-adapted tiered biopsy protocol

Based on the above evidence, a tiered biopsy optimization protocol is proposed, centered on PI-RADS category and integrating adjunctive parameters and lesion characteristics.

Tier 1 — Selective omission zone

Population: PI-RADS 4–5 with lesion ≥1 cm AND PSAD ≥0.20 ng/mL/cm³ (including large PI-RADS 4 lesions).

Strategy: targeted biopsy alone or targeted biopsy with ipsilateral/perilesional systematic biopsy. TB+PLB within a 10-mm perilesional penumbra is preferred when transperineal route is used.

Rationale: In this population, the incremental csPCa yield of systematic biopsy is low. Omission of contralateral and distant systematic cores reduces trauma and biopsy-related complications without substantially compromising significant cancer detection (20, 56). Recent RCTs [PMID: 40760321 (66), PMID: 38158249 (67)] demonstrate that TB+iSB is non-inferior to standard combined biopsy in this population. However, the 2026 network meta-analysis findings require that this recommendation be graded as Low evidence and applied cautiously. The term “selective” rather than “safe” is deliberately used to reflect the conditional nature of this recommendation and the limited long-term oncologic outcome data supporting de-escalation.

Tier 2 — Conditional optimization zone

Population: PI-RADS 3, small PI-RADS 4, borderline PSAD, or equivocal biomarker.

Strategy: perform secondary risk stratification using PSAD, SelectMDx or ExoDx if available, and zone-specific interpretation. If biopsy is required, use targeted biopsy plus six-core ipsilateral/perilesional systematic biopsy rather than whole-gland 12-core systematic biopsy. Biopsy avoidance may be considered in patients with negative confirmatory testing (e.g., PHI <27, PSMA SUVmax below threshold, negative SelectMDx/ExoDx).

Rationale: This strategy reduces unnecessary biopsies in low-risk patients while maintaining adequate csPCa detection in those who require histologic confirmation (21, 22, 31, 81).

Tier 3 — Standard coverage zone

Population: biopsy-naïve high-risk men, multifocal disease, suspected extraprostatic extension, young patients with high genetic risk, previous negative biopsy with rising PSA, or cases where maximal staging accuracy is required.

Strategy: targeted biopsy plus standard systematic biopsy.

Rationale: These patients have a higher risk of MRI-invisible significant disease or multifocal disease, and combined biopsy remains the safest approach (59, 80).

### 6.1 Precedence rules for tier allocation

When a patient meets criteria for more than one tier, the following precedence rules apply: (1) Tier 3 (Standard Coverage) always takes precedence over Tier 1 and Tier 2 when any of the following are present: multifocal disease (≥2 PI-RADS ≥3 lesions), suspected extraprostatic extension on MRI, prior negative biopsy with rising PSA, or young patients (<55 years) with high genetic risk. (2) Within Tier 1, biopsy-naïve status alone does not constitute a Tier 3 indication if the patient has a single PI-RADS 5 lesion ≥1 cm with PSAD ≥0.20 ng/mL/cm³ and no other high-risk features. (3) ‘High-risk features’ for Tier 3 assignment are specifically defined as: Gleason pattern 4 or 5 on prior biopsy, PSA velocity >2 ng/mL/year, family history of metastatic prostate cancer, or germline mutation carrier status. (4) In cases of genuine ambiguity, the more conservative strategy (Tier 3) should be selected.

The recommended stratified biopsy optimization protocol is summarized in **Table 2**. An operational decision matrix with quantified risks and benefits is provided in **Table 3**.

**Table 2:** PI-RADS-based biopsy decision recommendations.

PI-RADS category	PSAD and lesion characteristics	Suggested approach	Evidence level
PI-RADS 5	Large lesion (≥1 cm) + PSAD ≥0.20	TB + PLB (10 mm) or TB + ipsilateral SB	Low
	Large lesion (≥1 cm) + PSAD <0.15	TB + limited SB (6-core)	Low-Moderate
	Small lesion (<1 cm)	TB + standard SB	Moderate
PI-RADS 4	Large lesion (≥1 cm) + PSAD ≥0.20	TB + PLB	Moderate
	Small lesion (<1 cm) or PSAD <0.15	TB + limited or standard SB	Moderate
PI-RADS 3	PSAD <0.10–0.15 and low zone-specific risk	Defer or biomarker triage	Moderate
	PSAD high or biomarker positive	TB + 6-core/perilesional SB	Moderate
PI-RADS 1–2	Free of other risk indicators	Avoid biopsy	High

TB, targeted biopsy; PLB, perilesional biopsy; SB, systematic biopsy; PSAD, prostate-specific antigen density (ng/mL/cm³). Evidence levels assigned using GRADE methodology. Thresholds should be locally calibrated.

**Table 3:** Operational decision matrix with quantified risks and benefits.

PI-RADS	Clinical scenario	Route	PSAD	Lesion size	Strategy	Expected csPCa detection	Expected cisPCa overdetection	Evidence level
5	Biopsy-naïve	TP	≥0.20	≥1 cm	TB+PLB (10 mm)	82–87%	3–5%	Low
	Biopsy-naïve	TR	≥0.20	≥1 cm	TB+limited SB	84–89%	5–8%	Low
4	Biopsy-naïve	TP	0.15–0.20	≥1 cm	TB+PLB	55–65%	8–12%	Moderate
	Biopsy-naïve	TP	<0.15	<1 cm	TB+6-core SB	35–45%	15–20%	Moderate
3	Biopsy-naïve	TP	<0.10	Any	Defer + PSAD monitoring	5–8%	—	Moderate
	Biopsy-naïve	TP	0.10–0.15	Any	Biomarker triage	10–15%	10–15%	Moderate
	Prior-negative	TP	<0.13	Any	TB only	8–12%	5–8%	Moderate
	Active surveillance	Any	<0.10	Any	Defer repeat biopsy	3–5%	—	Low

csPCa, clinically significant prostate cancer (GG≥2); cisPCa, clinically insignificant prostate cancer (GG1); TB, targeted biopsy; PLB, perilesional biopsy; SB, systematic biopsy; TP, transperineal; TR, transrectal; PSAD, PSA density (ng/mL/cm³). Detection rates are approximate estimates derived from the cited literature and should be locally calibrated.

## 7 Limitations of current evidence

The literature has several important limitations:

1. Study design: Most available data are retrospective or nonrandomized prospective cohorts. Head-to-head randomized trials comparing targeted biopsy alone versus combined biopsy for csPCa detection are scarce (7, 69).
2. Population: Definitions of csPCa, systematic biopsy, and targeted biopsy vary across studies. The number of targeted cores, MRI fusion technique, and operator experience are frequently not standardized (82, 83).
3. Technical variability: PSAD threshold interpretation is affected by MRI-derived versus TRUS-derived prostate volumes, and many studies do not specify the method (69, 84).
4. Racial and ethnic gaps: Comparative data for Asian, African, and Caucasian populations are insufficient. African American-specific studies exist, but Asian-specific calibration is lacking (37, 74, 85).
5. Trial registry uncertainty: The data from NCT06584279 were obtained from the published abstract, AUA 2025 presentation, and subsequent peer-reviewed publication (Cheng et al., Cancer, 2026; PMID: 40760321 (66)). The original search did not identify these data because they were published after the search date. The revised manuscript incorporates these published data and clarifies the evidence source throughout (86). The comparable non-inferiority evidence for six-core versus 12-core systematic biopsy comes from NCT06684652 (55).
6. Short follow-up: Nearly all studies use csPCa detection rate as a surrogate endpoint; long-term oncologic outcomes are rarely reported (87).

## 8 Future directions

### 8.1 Randomized controlled trials

Large, multicenter, stratified randomized trials are required to test the proposed tiered approach. The available evidence from NCT06684652 supports non-inferiority of six-core versus 12-core systematic biopsy for csPCa detection (55), but this trial alone is insufficient to justify all de-escalation strategies.

### 8.2 Biomarkers and multimodal risk models

Future studies should evaluate whether biomarkers such as SelectMDx, ExoDx, Stockholm3, and MyProstateScore can be integrated into PI-RADS-based decision tools (88, 89). Specifically, head-to-head comparisons of SelectMDx versus ExoDx versus Stockholm3 in the PI-RADS 3 subgroup are needed to guide test selection (15, 90). PSMA PET/CT may also improve triage of PI-RADS 3 lesions because it has high specificity and negative predictive value (39, 91).

### 8.3 Long-term endpoints

Future studies should move beyond csPCa detection rate and report: Adverse pathology at radical prostatectomy; Biochemical recurrence; Metastasis-free survival; Overall survival; Focal therapy eligibility; Patient-reported outcomes and quality of life. If these endpoints are not evaluated, the long-term safety of de-escalation will remain incompletely defined (92).

### 8.4 Health services and cost outcomes

Few studies address the health-economic implications of reduced biopsy cores and omitted systematic biopsies. Future research should include cost-effectiveness analysis from both patient and system perspectives (93).

**Figure 2** illustrates a forward-looking integrated workflow for next-generation prostate biopsy. The pipeline begins with mpMRI acquisition, followed by registration and fusion with real-time transrectal ultrasound (TRUS) for accurate 3D lesion localization. AI-assisted real-time precision puncture guidance then directs both targeted and systematic sampling. Notably, this system incorporates a closed-loop feedback mechanism: pathological outcomes are fed back into a multimodal prognostic prediction model, enabling continuous refinement of the biopsy protocol. This architecture exemplifies how AI, advanced image fusion, and predictive modeling may converge to create a learning healthcare system that not only optimizes current biopsy decisions but also iteratively improves future performance.

**Figure 2:**
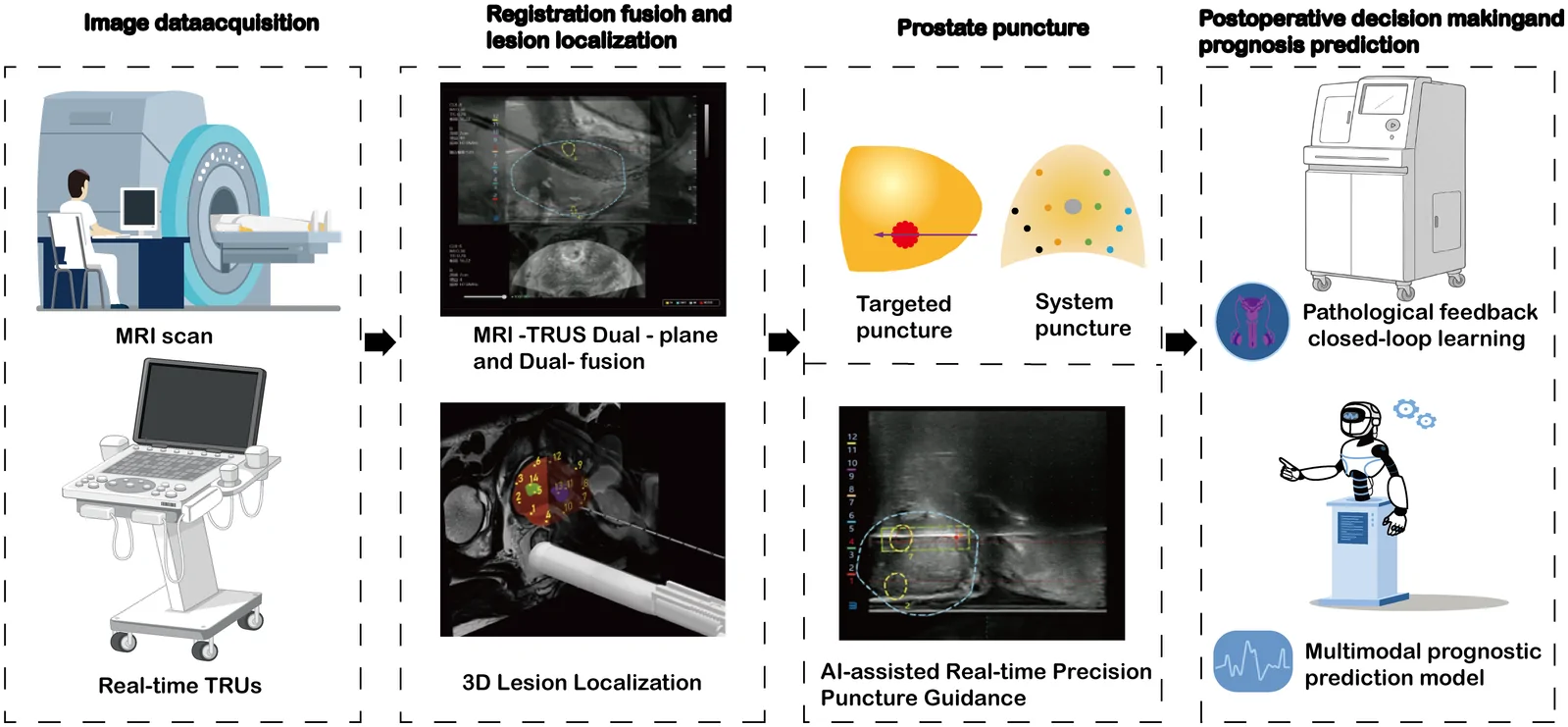
Future integrated workflow for AI-enhanced prostate biopsy. The pipeline comprises five core components: (1) mpMRI acquisition for lesion detection; (2) image registration and fusion with real-time TRUS for accurate 3D lesion localization; (3) AI-assisted real-time precision guidance for both targeted and systematic puncture; (4) multimodal prognostic prediction model integrating pathological and imaging data; and (5) closed-loop feedback learning, where pathological outcomes are used to refine the prediction model and guide future biopsy decisions. This conceptual framework illustrates the potential of a learning healthcare system to continuously improve biopsy precision and risk stratification.

However, several challenges remain. Current AI sensitivity at the lesion level (80%) remains lower than that of radiologists (93%) (30, 94), suggesting AI is better suited as a quality assurance tool rather than a complete replacement for human reading. Standardization of AI algorithms across different MRI platforms and validation in diverse populations are essential before widespread clinical adoption (30).

## 9 Summary and clinical recommendations

This review supports the conclusion that prostate biopsy should transition from a uniform combined approach to a risk-adapted strategy based on PI-RADS classification, PSAD, lesion characteristics, and biomarkers. However, de-escalation is not universally safe.

The key recommendations are:

1. PI-RADS remains the initial stratification tool, but it should not be used alone.
2. For PI-RADS 5 lesions—especially large lesions or those with high PSAD—targeted biopsy alone or targeted biopsy with ipsilateral/perilesional systematic biopsy may be considered cautiously, with Low evidence grading.
3. For PI-RADS 4 lesions, current evidence does not support routine omission of systematic biopsy; combined biopsy is preferred, although limited systematic sampling may be acceptable in selected patients.
4. For PI-RADS 3 lesions, PSAD thresholds should be applied with caution. The commonly cited 0.15 ng/mL/cm³ cutoff can assist triage, but lower thresholds of 0.10–0.13 ng/mL/cm³ improve safety at the cost of avoiding fewer biopsies. The method of prostate volume measurement must be specified. Scenario-specific thresholds for transperineal versus transrectal route and peripheral versus transition zone are provided in Section 5.2.1.
5. In PI-RADS 3 patients with borderline PSAD, urinary biomarkers such as SelectMDx or ExoDx should be considered. Stockholm3 lacks specific PI-RADS 3 validation.
6. For patients requiring biopsy in the conditional optimization zone, targeted biopsy plus six-core ipsilateral/perilesional systematic biopsy is acceptable and may reduce morbidity.
7. Transperineal biopsy is preferred when anterior or apical lesions are suspected and when reducing infection risk is a priority. Route-specific recommendations are detailed in Section 5.4.1.
8. PI-RADS 1–2 patients should generally avoid biopsy unless additional indicators are present.
9. These recommendations are conditional and should be calibrated to local radiology and pathology performance.

The long-term objective of precision prostate biopsy is not simply to detect more cancer, but to identify and treat clinically significant prostate cancer while minimizing harm from overdiagnosis and unnecessary biopsy. Achieving this will require better integration of imaging, clinical parameters, molecular biomarkers, and patient-level decision-making.

## Glossary

ADC: Apparent Diffusion Coefficient
AI: Artificial Intelligence
AUC: Area Under the Curve
AUROC: Area Under the Receiver Operating Characteristic Curve
BpMRI: Biparametric Magnetic Resonance Imaging
CEUS: Contrast-Enhanced Ultrasound
CHAID: Chi-squared Automatic Interaction Detector
ciPCa: Clinically Insignificant Prostate Cancer
csPCa: Clinically Significant Prostate Cancer
CT: Computed Tomography
DCE: Dynamic Contrast-Enhanced
DICOM: Digital Imaging and Communications in Medicine
DWI: Diffusion-Weighted Imaging
EAU: European Association of Urology
EPI: ExoDx Prostate IntelliScore
f/tPSA: Free-to-Total Prostate-Specific Antigen Ratio
GG: Grade Group
GRADE: Grading of Recommendations Assessment, Development and Evaluation
HR: Hazard Ratio
iSB: Ipsilateral Systematic Biopsy
ISUP: International Society of Urological Pathology
mpMRI: Multiparametric Magnetic Resonance Imaging
MRI: Magnetic Resonance Imaging
NCT: National Clinical Trial
NPV: Negative Predictive Value
OR: Odds Ratio
PET: Positron Emission Tomography
PHI: Prostate Health Index
PI-RADS: Prostate Imaging Reporting and Data System
PPV: Positive Predictive Value
PRECISE: Prostate Cancer Magnetic Resonance Imaging Study (trial acronym)
PRIAS: Prostate Cancer Research International: Active Surveillance (study acronym)
PSA: Prostate-Specific Antigen
PSAD: Prostate-Specific Antigen Density
PSMA: Prostate-Specific Membrane Antigen
PV: Prostate Volume
PZ: Peripheral Zone
SB: Systematic Biopsy
SEPTA: Stockholm3 Multiethnic Trial (trial acronym)
STHLM3: Stockholm3 (prostate cancer risk score)
SUVmax: Maximum Standardized Uptake Value
T2WI: T2-Weighted Imaging
TB: Targeted Biopsy
TP: Transperineal
TRUS: Transrectal Ultrasound
TZ: Transition Zone
