# Supplementary material for: Targeted biopsy with or without systematic biopsy in PI-RADS-stratified prostate biopsy: a risk-adapted narrative review and clinical proposal
Source: Front Oncol. 2026 Oct 26;16:1996528. doi: 10.3389/fonc.2026.1996528 (PMC13746171; doi:10.3389/fonc.2026.1996528)
Supplement: Supplementary file 1 [file Table1.docx]

**Supplementary Table S1. Evidence map of major studies included in the narrative synthesis**

| Study (year) | Design | PI-RADS category | Key finding | Evidence level |
| --- | --- | --- | --- | --- |
| Hugosson et al. (2022) | RCT | All | MRI-targeted biopsy alone reduced ciPCa detection by 54% vs SB | High |
| Klotz et al. (2024) PRECISE | RCT | All | MRI-targeted biopsy non-inferior to SB for csPCa; 38% avoided biopsy | High |
| Ahdoot et al. (2025) TRIO | Prospective cohort | PI-RADS 4–5 | PSAD ≥0.2: incremental csPCa yield of SB only 2% | Moderate |
| Cheng et al. (2026) NCT06584279 | Multicenter paired non-inferiority | PI-RADS ≥4 or PI-RADS 3 + PSAD ≥0.15 | TB+iSB non-inferior to TB+SB (40.78% vs 42.38%) | Moderate |
| Nature Communications NMA (2026) | Network meta-analysis | PI-RADS 5 | Omitting SB in PI-RADS 5 not supported | Moderate |
| MIRAGE (2026) | Prospective | PI-RADS 4–5 | TB+PLB makes distant SB unnecessary | Moderate |
| ProBIOPSY consensus (2026) | Consensus | PI-RADS 4–5 | TB+PLB within 10-mm penumbra sufficient | Moderate |
| PERI-PRO (2025) | RCT | PI-RADS 4–5 | TPLBx non-inferior to CTSBx with better safety | Moderate |
| MR-PROPER (2026) | Prospective multicenter | PI-RADS 3 | PSAD ≥0.20 avoided 29% biopsies, missed 1.3% GG≥2 | Moderate |
| Buhas et al. (2026) | Multicenter cohort | PI-RADS 3 | PSAD-based omission of systematic cores feasible | Moderate |
| Wang et al. (2025) | Nomogram | PI-RADS 4–5 | PHI + PSMA SUVmax reduced biopsies by 92.41%, missed 1.53% csPCa | Low |
| SEXTANT-PRO (2025) | RCT | PI-RADS 3 | TB+6SB non-inferior to TB+12SB | Moderate |
